# Toll-like receptor 4: innate immune regulator of neuroimmune and neuroendocrine interactions in stress and major depressive disorder

**DOI:** 10.3389/fnins.2014.00309

**Published:** 2014-09-30

**Authors:** JiaJun Liu, Femke Buisman-Pijlman, Mark R. Hutchinson

**Affiliations:** ^1^Neuroimmunopharmacology Group, Discipline of Physiology, School of Medical Sciences, The University of AdelaideAdelaide, SA, Australia; ^2^Discipline of Pharmacology, School of Medical Sciences, The University of AdelaideAdelaide, SA, Australia

**Keywords:** toll-like receptor 4, TLR4, HPA, neuroendocrine, neuroimmunology, stress, depression, MDD

## Abstract

Major depressive disorder (MDD) poses one of the highest disease burdens worldwide. Yet, current treatments targeting serotonergic and noradrenaline reuptake systems are insufficient to provide long-term relief from depressive symptoms in most patients, indicating the need for new treatment targets. Having the ability to influence behavior similar to depressive symptoms, as well as communicate with neuronal and neuroendocrine systems, the innate immune system is a strong candidate for MDD treatments. Given the complex nature of immune signaling, the main question becomes: What is the role of the innate immune system in MDD? The current review presents evidence that toll-like receptor 4 (TLR4), via driving both peripheral and central immune responses, can interact with serotonergic neurotransmission and cause neuroendocrine disturbances, thus integrating with widely observed hallmarks of MDD. Additionally, through describing the multi-directional communication between immune, neural and endocrine systems in stress, TLR4—related mechanisms can mediate stress-induced adaptations, which are necessary for the development of MDD. Therefore, apart from exogenous pathogenic mechanisms, TLR4 is involved in immune changes as a result of endogenous stress signals, playing an integral part in the pathophysiology, and could be a potential target for pharmacological treatments to improve current interventions for MDD.

## Introduction

Major depressive disorder (MDD) represents a combination of disturbances to mood, cognition, sleep and appetite, which causes impairment to individual functioning lasting a minimum of 2 weeks (American Psychiatric Association, 2013). MDD consistently ranks within the top 4 highest “years lived with disability” (Vos et al., 2012), and accounts for 7.4% of total disability-adjusted life years worldwide (Whiteford et al., 2013). In addition, depressive disorders have an estimated 93.5% comorbidity with other diseases, most commonly with chronic pain, anxiety, bipolar disorder, post-traumatic stress disorder, diabetes and neurological disorders (Gadermann et al., 2012). Major depression thus bears one of the highest disease burdens, with matching economic and societal costs. Yet, current pharmacological treatments using serotonin and noradrenaline reuptake inhibitors (SSRI and SNRI, respectively) are inefficient, requiring to treat 7 patients in order to gain one positive outcome (Arroll et al., 2009). This suggests that alterations to the serotonergic pathway are only partly responsible for MDD, and other mechanisms must be involved. Thus, it is sensible to study other systems, in order to improve current treatments for MDD.

Ever since early observations of increased immune markers in psychiatric patients, the immune system has become increasingly associated with various psychosomatic illnesses (Solomon et al., 1969). In the case of MDD, patients exhibit increased circulating peripheral cytokines, which are immune signaling molecules that can be pro or anti-inflammatory (Anisman and Hayley, 2012; Lichtblau et al., 2013). Additionally, a decrease in depressive symptoms is coupled with a normalization of immune signaling levels (Gazal et al., 2013), suggesting that there may be immune involvement in MDD.

## Neuroinflammatory events: neuroinflammation and neurokine signaling in MDD

Although immune signaling in the brain is comprised of signals from resident glial cells, peripheral to central immune communications and migration of peripheral cells into central compartments, it is important to note the phenotypic differences between different neuroinflammatory diseases. The term “Neuroinflammation” is commonly used to encompass increased immune activation in the CNS. However, the central nervous system (CNS) can take on different inflammatory states, and there is a distinction between the magnitude of immune responses in the CNS by varied causes. For example, neurodegenerative disorders including Alzheimer's and Parkinson's disease are characterized by widespread immune signaling in the CNS, oxidative stress, and increased immune trafficking into the brain, resulting in systemic inflammation accompanied with progressive damage (Heneka et al., 2014). On the other hand, acute and sub-maximal immune challenges such as that triggered by opioid exposure can also cause increased central immune signaling, but the resulting inflammatory response is of a much lower magnitude (Stevens et al., 2013). This sub-inflammatory state is attributed to the direct actions of opioids on CNS expressed toll-like receptor 4 (TLR4), since opioids readily cross the BBB, rather than the indirect peripheral to central immune response following bacterial infections (Hutchinson and Watkins, 2014; Jacobsen et al., 2014).

Classically, inflammation involves swelling, heat, and pain, coupled with a coordinated infiltration of various immune cells into the affected area. In many neurological conditions, this large-scale damage is not seen unless in terminal stages, or in cases of major BBB compromise. Thus, the use of “neuroinflammation” to refer to central immune activity can become confusing, and a clear distinction between high magnitude and submaximal immune states is required.

Neurokines refer to neurally active cytokines (Nathanson, 2012), which can be upregulated as a result of peripheral infections or innate immune activation without the infectious agent crossing the BBB (McCusker and Kelley, 2013). An important differentiation between increased neurokine signaling and neuroinflammation is the reversibility of the effects. Central cytokine increase resulting from low-grade infections, exercise and stress result in reversible neuronal changes (McCusker and Kelley, 2013). These neuronal changes include the upregulation of AMPA and NMDA receptors, as well as decreased expression of GABA receptors on neurons by cytokines IL-1β and TNF-α, causing reversible increased excitation (see Viviani et al., 2014 for a review). We thus propose the use of the terms “increased neurokine signaling” or “increased central immune signaling” to apply to these sub-inflammatory states, and only when there is large-scale damage as a result of immune cell derived neurotoxicity and inflammation should the term “neuroinflammation” be applied in order to reduce confusion within the literature.

In regards to MDD, current evidence indicates a milder immune signaling phenotype more akin to increased neurokine signaling, rather than neuroinflammation. Compared with the continued accumulated loss of function observed in neurodegenerative disorders such as Alzheimer's and Parkinson's disease, MDD has periods of active disease characterized by depressive episodes followed by remission. During remission, peripheral blood expression of TNF superfamily 12–13 mRNA is lower than in active disease (Otsuki et al., 2010). This indicates that the immune signaling is influenced by the state of the disorder, but critically, the degree of immune signaling required for the presentation of a disease symptom may be significantly higher than that needed for MDD to be observed, as illustrated by the development of depressive-like behavior prior to the development of significant demyelination in experimental autoimmune encephalitis (Acharjee et al., 2013). Furthermore, structural changes in hippocampal volume measured using MRI appear reversible in patients with MDD (Frodl et al., 2004). Antidepressants (Horikawa et al., 2010; Alboni et al., 2013; Obuchowicz et al., 2014) and cognitive-behavioral therapy (Gazal et al., 2013) are also able to reduce cytokine expression in depressive disorders. Taken together, there is strong evidence for the reversibility of inflammatory markers that closely relates to the state of depressive symptoms. Along with the bi-directional neuroimmune connection, this reversibility and liability of the condition suggests that targeting the central immune system could be a promising treatment option for MDD.

## The role of toll-like receptor 4 in MDD

Recently, TLR4 has come to the forefront of research linking neuroimmune signaling and MDD, through driving immune to brain communication. TLR4 is an innate immune pattern recognition receptor, which is part of the Interleukin-1 Receptor/Toll-Like Receptor Superfamily containing a toll-like/IL-1 Receptor (TIR) domain and Leucine-rich repeat motif in the extracellular domain. TLR4 recognizes endogenous danger associated molecular patterns (DAMPs) including heat shock proteins (HSP) and high mobility group box 1 (HMGB1), exogenous pathogen associated molecular patterns (PAMPs) such as lipopolysaccharide (LPS), as well as microbiome/microbe associated molecular patterns (MAMPs) (Akira and Takeda, 2004). Unlike other toll-like receptors, activation of TLR4 triggers pro-inflammatory transcription via 2 adaptor proteins, Myeloid differentiation primary response 88 (MyD88) and TIR domain-containing adapter inducing IFN-β (TRIF), which induces transcription factors NF-κB, AP-1, and IRF3 (Xu et al., 2000; Watters et al., 2007). Activation of these transcription factors causes the production of pro-inflammatory cytokines including IL-1β, TNF-α, IL-6, and CXCL10, along with an upregulation of proteins including cyclooxygenase 2 (COX-2), resulting in pro-inflammatory signaling (Akira and Takeda, 2004).

In the CNS, TLR4 is predominantly expressed on microglia, and is also to a lesser extent expressed on neurons (Zhao et al., 2014). Through modulation of neuroimmune activity, TLR4 is implicated in various neuropathological conditions that afflict the CNS, including neuropathic pain (Lewis et al., 2012) and neurodegenerative diseases (Heneka et al., 2014). Increasingly, these disorders are recognized as existing on a spectrum from altered neuroimmune signaling (e.g., neuropathic pain) through to gross neuroinflammation (e.g., Neurodegenerative diseases).

MDD, being increasingly classified as a neuroimmune disorder, may also have TLR4 involvement (Gárate et al., 2011; Hines et al., 2013). Recent evidence has found that peripheral blood mononuclear cells (PBMC) of patients with MDD express higher levels of TLR4 (Kéri et al., 2014). Importantly, the authors showed that this heightened expression was reduced following treatment, and paralleled improvement in depressive symptoms. This responsiveness toward depressive states indicates that TLR4 activity could directly be involved in the pathophysiology of MDD.

Given the involvement of the immune system in numerous neurological disorders, it is now accepted that the CNS is not an immune privileged organ that exists in isolation of the immune system. The CNS can no longer be thought of as resistant to immune signals, or protected from immune damage (Ransohoff and Brown, 2012). Rather than separate entities, immune, neuronal and endocrine systems appear to be in constant bidirectional communication and a dysregulation of these systems can result in pathological states within the CNS (see Kelley and McCusker, 2014 for review). The current review thus posits that TLR4, being central to the immune to brain, and immune to neuroendocrine communication, underlies the neuroimmune signaling events observed in the pathophysiology of stress-related disorders including MDD, across systemic and cellular levels.

### TLR4 activity can influence behavior and MDD

Using LPS as an agonist, peripheral TLR4 activation is sufficient to cause changes in motivational state and can trigger sickness behavior (Hines et al., 2013). Sickness behavior is characterized by increased anhedonia, lethargy, loss of locomotion and anorexia following immune challenges (Dantzer and Kelley, 2007). Once thought to stem from altered energy balance, it is clear that these immune signaling initiated behavioral adaptations are driven by specific cytokine-dependent signaling cascades (Dantzer, 2004). Thus, an immune stimulus can drive complex higher order behavioral adaptations, providing direct evidence for the link between the immune system and symptoms of depression.

Although the idea of sickness behavior may appear surprising since pathogens themselves are imperceptible by our classic sensory organs, it makes sense that the body needs to know when it encounters pathogens in order to make behavioral adjustments. Through reduction in locomotion, sickness behavior can promote recovery from the immune challenges. Sickness behavior can therefore be considered as adaptive since it promotes homeostasis within the conditions of the stressor (Dantzer and Kelley, 2007).

The effect of peripheral immune activation on behavior is thought to be mediated by three main mechanisms: (1) peripheral release of pro-inflammatory cytokines can cross the blood brain barrier (BBB) via “leaky” subventricular organs, thus directly increasing neurokine signaling, (2) These activated immune cells can also cross the BBB directly to cause neuroimmune signaling, and (3) peripheral cytokines can also stimulate afferent pathways such as the vagus nerve, causing behavioral changes via neural mechanisms (see McCusker and Kelley, 2013 for review). Besides these mechanisms, emerging evidence have demonstrated that monocytes can adhere and roll along the cerebral vasculature, and can cause increased central immune signaling without crossing the BBB, and this is associated with increases in inducible Nitric Oxide Syntase (D'Mello et al., 2013). The authors further showed that inhibiting this mechanism could improve sickness behavior as a result of peripheral inflammation, thus suggesting another mode of peripheral to central communication with behavioral implications.

Importantly, the trigger for behavioral changes need not originate from pathogenic mechanisms, since cytokine administration is sufficient to elicit sickness behavior similar to bacterial stimulation (Tazi et al., 1988; Dantzer, 2004). This means that other potential sources of immune activation, including endogenous danger signals, or neurogenic activation could trigger this response.

Strong parallels can be drawn between sickness behavior and depressive behavior, namely reduced locomotion, anhedonia, and dysregulated sleep and food intake (McCusker and Kelley, 2013). Since MDD can be chronic and recurring, the question arises whether patients with MDD are just displaying chronic dysregulation in inflammation or immune signaling? Indeed, patients suffering from MDD display heightened circulating cytokine levels, indicative of increased immune signaling (Anisman and Hayley, 2012; Lichtblau et al., 2013). Furthermore, there is some evidence showing that celecoxib, an NSAID that inhibits COX-2, can improve depression scores as well as increase remission rates in patients receiving anti-depressants (Faridhosseini et al., 2014; Na et al., 2014). In rodent models, NSAID administration can improve depressive-like behavior, measured using the forced swim test and tail suspension tests (Maciel et al., 2013; Guan et al., 2014). Notably however, these studies indicate that the anti-depressive effect of NSAID requires a higher dose in order to achieve comparable results to SSRIs, and are more effective in immune stressor induced depressive-like behavior. Additionally, there is some debate about the efficacy of these treatments since NSAIDs can also reduce efficacy of SSRI treatment when given in conjunction, in both animals and patients (Warner-Schmidt et al., 2011). MDD thus appears to have an immune component, but the exact mechanisms behind this interaction require more investigation.

### Cell mediated mechanisms of central immune signaling events associated with MDD

It has been confirmed that both peripheral and central administration of LPS can induce sickness behavior, showing that both peripheral and central immune signaling are involved in perpetuating this behavioral change (Huang et al., 2008; Hines et al., 2013). This TLR4 mediated Innate immune signaling in the CNS is mainly undertaken by 2 cell populations, the resident glial cells, and infiltrating peripheral immune cells.

Glial cells, consisting of oligodendrocytes, astrocytes and microglia, vastly outnumber neurons by an estimated 10–50-fold (Temburni and Jacob, 2001; Banati, 2003). These cells were previously regarded as inert support cells that do not directly influence neurotransmission. However, there is increasing evidence that glial cells are integral to both physiological functions and pathophysiological states in the CNS.

#### Astrocytes

Astrocytes are the most abundant cell type in the CNS, providing structural and trophic support to neurons. They have star-shaped morphology, with processes that can be in contact with up to 100,000 neurons (Halassa et al., 2007). Although astrocytes are not capable of producing action potentials, neurotransmitter binding to receptors present on astrocytes can induce Ca2+ waves (Sharma and Vijayaraghavan, 2001; Schipke et al., 2011). In addition to forming close appositions with synapses, astrocytes can influence the chemical environment through secretion of various compounds such as ATP, adenosine and acetylcholine, a process known as gliotransmission. Astrocytes can therefore influence neurotransmission on a synaptic level and could play a role in aggregating neural responses. Collectively, astrocyte involvement with synaptic activity is termed the tripartite synapse (Araque et al., 2014).

Across the CNS, astrocytes display different phenotypes and can adopt different states (Sun and Jakobs, 2012). Activated astrocytes display less branching morphological characteristics, and this is associated with glial fribrillary acid protein (GFAP) upregulation (Pekny and Nilsson, 2005). Activated in injury, astrocytes can be both protective by releasing factors facilitating recovery (Gimsa et al., 2013), and disruptive to neuronal functioning via inhibiting axon growth through formation of glial scars after extended activation (Smith-Thomas et al., 1994; Yuan and He, 2013). Moreover, radial glia located at the subventricular zones also express GFAP, but act as precursor cells capable of differentiating into neurons and astrocytes, which can migrate to several areas including the cortex, displaying ability for adult neurogenesis and cell renewal (Sundholm-Peters et al., 2005). The role of astrocytes is therefore varied and important to normal physiology, but astrocytes can also participate in both neuroimmune signaling and neuroinflammatory disease progression.

In MDD, astrocytes may be involved in the progression of the disease through participating in the reuptake of serotonin. In the presence of TNF-α, serotonin transporters expressed on astrocytes increase reuptake of serotonin in a dose-dependent manner, and this effect is attenuated by administration of SSRIs (Malynn et al., 2013). Interestingly, SSRI are also able to influence astrocytic Ca2+ waves in a similar way to serotonin administration (Schipke et al., 2011). Although unclear whether astrocytes are an integral target for anti-depressant treatment, it appears that astrocytes can play a role in serotonin neurotransmission, and therefore be involved in the pathophysiology of MDD.

#### Microglia

Microglia are the resident immunocompetent cells in the CNS, and play an active role in neuroinflammatory actions by release of cytokines, chemokines phagocytosis and removal of debris, directly modulating neuroimmune activity. Microglia are thus the main cell type investigated in neuroimmune signaling events and neuroinflammatory/neurodegenerative conditions. Being implicated in synaptic pruning during CNS development, microglia not only perpetuate damage, but also are integral for normal functioning (Schwartz et al., 2013). Microglia too have different activation states, commonly referred to as M1 and M2, the pro and anti-inflammatory phenotypes, respectively (Olah et al., 2011). When activated to an M1 phenotype, microglia display a more ameboid morphology, and secrete cytokines and chemokines to signal for other immune cells. Microglia can also function as antigen presenting cells through MHC-II expression, therefore possessing the ability to trigger the adaptive immune responses within the CNS (Harms et al., 2013). Expressing pattern recognition receptors such as TLR4, microglia are responsive to DAMPs, MAMPs, and PAMPs. Moreover, TLR4 activation can shift microglia toward a M1 phenotype, inducing pro-inflammatory responses in the CNS (Ajmone-Cat et al., 2013).

Besides functioning as an antigen-presenting cell, microglia can influence neuronal functions through expression of glutamate transporters (Persson et al., 2005), but to a lesser extent as compared to astrocytes (Beschorner et al., 2007). In addition, microglia in the “resting” state actively survey the surrounding area for chemical changes, and can rapidly respond to stimuli (Nimmerjahn et al., 2005). Thus, even during an immunologically dormant state, microglia are able to influence neurotransmission and can rapidly respond to danger signals. Aiding this responsiveness are the toll-like receptors, which can recognize multiple stimuli and modulate the activation states of microglia.

Microglia, through TLR4-dependent signaling (Hines et al., 2013), and production of cytokines (Henry et al., 2009; Dobos et al., 2012), are regarded as mediators of central immune signaling in animal models of sickness behavior. In addition, changes in microglial reactivity states are also associated with the induction of stress-induced depressive like behavior (Pan et al., 2014). In a recent study, minocycline, an antibiotic that has also shown the ability to suppress central immune signaling by acting on microglia, can prevent the development of depressive-like behavior, tested using sucrose preference and social exploration (Kreisel et al., 2014). This reduction of depressive-like behavior was found in conjunction with changes to microglia morphology and proliferation. On the other hand, the study illustrated that simply inhibiting microglia chronically would not be a viable treatment option, since microglial activation states change from an initial proliferative state to later decline from acute to chronic models, and central immune suppression exacerbates depressive-like behavior in a chronic model. Instead, the authors proposed that depression is related to either an over or under activation of microglia, and treatments should strive toward a balance in activation states. Taken together, microglia appear important in central immune signaling and immune to brain communication in MDD, but this relationship is not is not uni-directional, and appears to be time-dependent.

#### Peripheral immune cells and their actions on the CNS

Although the CNS is protected from many peripheral factors by the BBB, peripheral immune cells have been found to infiltrate and drive inflammation within the CNS (Schweingruber et al., 2014; Vogel et al., 2014). Perivascular macrophages and circulating monocytes can cross the BBB into the CNS through the expression of adhesion molecules such as ICAM and VCAM on the endothelium of blood vessels surrounding the CNS (Wong et al., 2007). Additionally, peripheral leukocytes can also roll and adhere to the cerebral vasculature, and cause increased central immune signaling without entering the CNS, thus communicating across the BBB (D'Mello et al., 2013). Glial cells themselves can also release chemokines such as CCL2, which can trigger peripheral immune cell extravasation into CNS tissue (Williams et al., 2013; Shieh et al., 2014). Thus, the central immune system can actively signal for peripheral immune cells to cross the BBB.

Contrary to other models of neuroinflammatory/neurodegenerative disorders Wohleb et al. (2014) have recently shown that peripheral bone marrow-derived cell infiltration is not just evident in models of BBB compromise, but can also occur in sub-inflammatory conditions as well. Interestingly, increased infiltration of peripheral monocytes can influence anxiety-like behavior, once again illustrating the ability for immune signaling to influence higher-order CNS function (Wohleb et al., 2014). This result, however, still needs more investigation, as it is widely held that immune-cell infiltration is a sign of neuroinflammation, and is not evident in sub-maximal levels of central immune signaling.

There is little direct evidence showing peripheral immune cell migration in MDD due to the lack of a representative animal model of MDD. Nevertheless, social defeat and psychological stress can trigger increased trafficking of peripheral cell infiltration (Brevet et al., 2010; Wohleb et al., 2013). Given that MDD is considered a stress disorder (for more information, see Section “what does stress have to do with it?”), this indicates that peripheral cell infiltration could be involved in MDD. However, isolating the exact cause of immune signaling in MDD is challenging, and the extent of peripherally driven immune responses in MDD patients is thus still unknown.

### Effect of increased immune (TLR4) activity on CNS neurotransmitter activity

So far, the best-characterized neuropathophysiology of MDD within the CNS is the dysregulation in serotonin neurotransmission, but the exact cause of this alteration is still unclear. There is growing evidence that glia are able to influence neurotransmission, and through these mechanisms, glia can contribute to the neuronal adaptations in MDD (Burke et al., 2014; Kreisel et al., 2014). Through close appositions with synapses in tripartite and tetrapartite arrangements, glia have access to the chemical environment of the synapse. Functionally, astrocytes can contribute to glutamate homeostasis by clearing excess glutamate from synapses. During situations of increased neuroimmune signaling, this process is impaired due to the down regulation of astrocyte glutamate transporters (EAAT1 and EAAT2), causing glutamate neurotoxicity and subsequent neuronal death (Tilleux and Hermans, 2008; Persson et al., 2009; Fang et al., 2012).

In relation to MDD, glia express serotonin transporters (Malynn et al., 2013), and can also directly inhibit serotonin production during neuroinflammation through the production of indoleamine-2,3-dioxygenase (IDO). IDO further interferes with the synthesis of serotonin by catalyzing tryptophan, forming quinolinic acid and 3-hydroxy-kynurenine, which can further result in neurotoxicity (O'Connor et al., 2009). Through this pathway, the increase in IDO reduces serotonin signaling as seen in MDD by impairing serotonin production, and can also cause direct damage to serotonergic neurons. Indeed, patients with MDD display and increased circulating kynurenine to tryptophan ratio, suggesting increased IDO activity (Quak et al., 2014). Moreover, this effect is thought to mediate depressive like behavior as a result of immune activation, as pharmacological inhibition of IDO is able to attenuate the increase in central immune signaling and depressive-like behavior in response to LPS administration in rodant models (Corona et al., 2010; Dobos et al., 2012). The ability of glia to influence serotonergic neurotransmission illustrates that rather than replace earlier notions of what causes depressive symptoms—that is, an impairment in serotonin metabolism—neuroimmune mechanisms instead contribute to and supplement neural mechanisms of disease.

### Effect of MDD treatments on immune signaling

The immune to brain communication is not uni-directional, since neuronal functions can also influence the activity of the immune system. This is especially evident in current treatments of MDD using SSRIs or SNRIs that work via alterations to serotonin and noradrenaline, respectively. Anti-depressants have shown to reduce LPS induced peripheral IL-6 and TNF-α production (Manikowska et al., 2014). SSRI administration can also attenuate CRH, TNF-α, and IL-1β mRNA expression in the hypothalamus after chronic treatments (Alboni et al., 2013). Glia are responsive to anti-depressant treatments, since SSRIs can decrease gliotransmission (Dhami et al., 2013), and can partially attenuate microglial secretion of TNF-α in response to LPS. Pharmacologically blocking the reuptake of serotonin can also reduce microglial reactivity and inhibit LPS-induced changes in microglia morphology (Horikawa et al., 2010; Obuchowicz et al., 2014). In addition, there is also evidence that antidepressants are protective against microglial- (Zhang et al., 2012) and MPTP-induced neurotoxicity (Chung et al., 2011). Thus, alterations to serotonergic neurotransmission can also influence glial and central immune activity, and this may contribute to the anti-depressive effects of SSRI. Together with evidence of immune modulation of neurotransmission, this illustrates the bi-directional communication between the neural and immune systems in both normal and pathophysiology.

## What does stress have to do with it?

Stress refers to a challenge to the body's homeostatic state, and can be classified broadly as psychological, physiological and immunological in origin. According to the diathesis-stress model of depression, stress is essential to the development of MDD, as stress is required in order to unmask the underlying individual predisposition to the disorder (Monroe and Simons, 1991). Biological and environmental factors thus interact to produce the physiological and psychological depression phenotype. Stress, regardless of type, activates the hypothalamus pituitary and adrenal (HPA) axis, which forms the neuroendocrine stress response. The HPA axis is therefore the most investigated link between stress and MDD. Furthermore, TLR4 activation is considered an immunological stress, and recent research demonstrates that it is deeply intertwined with the neuroendocrine stress response. Given the innate immune component of MDD as outlined earlier, we propose that the role of TLR4 in MDD is mediated at least in part by its interaction with the HPA axis.

### The classical HPA axis

When activated, the HPA axis works to restore homeostasis following different stressors (Chrousos, 2009). Activation of the HPA axis begins with neurons in the paraventricular nucleus of the hypothalamus (PVN), which secrete corticotropin releasing hormone (CRH). CRH then stimulates the pituitary gland to release adrenocorticotropic hormone (ACTH) into the blood circulation. Upon binding to melanocortin 2 receptor (MC2R) expressed on the zona fasciculata layer of the adrenal cortex, ACTH stimulates glucocorticoid (GC) production *de novo*. GC binds to cytoplasmic glucocorticoid receptors (GR) and mineralocorticoid receptors (MR) at a higher affinity (Sapolsky et al., 2000). Due to this higher affinity, the actions of baseline GC are thus associated with MR binding, whereas GR actions are attributed to upregulated GC by stressors.

GR binding causes receptor translocation into the nucleus through the aid of chaperone proteins such as heat shock protein 70 and 90 (HSP70, HSP90, respectively), where it can either dimerise and bind to glucocorticoid response elements (GRE) on DNA to either increase transcription of glucocorticoid responsive genes, or interact with other proteins such as transcription factors NF-κB and AP-1 (see Silverman and Sternberg, 2012 for review). GC actions are therefore extremely complex. Furthermore, most cells express GR, and thus GCs can influence the functions of multiple systems through diverse endocrine actions. GCs are also able to cross the BBB to influence CNS function including feeding back upon its own secretion by signaling via GR in the hippocampus to increase GABAergic tone on the PVN.

### HPA activity alterations in MDD

The HPA response is markedly changed during depression. Specifically, patients with MDD exhibit heightened cortisol levels in the morning, and possess a flatter diurnal slope throughout the day (Dinan, 1994; Jarcho et al., 2013). This indicates that a dysregulation in HPA activity is involved in the pathophysiology of depression, and further raises important questions as to what causes this HPA dysregulation, as well as how this fits in with the immune adaptations outlined earlier.

The hypersecretion of cortisol could mean either a problem with the feed-forward secretory pathway, or the negative feedback loop within the HPA axis. The former is unlikely true, as adrenal responsiveness to ACTH is normal in patients with depression (Rubin et al., 2006), showing that adrenal function itself is not altered in depressive disorders. On the other hand, patients exhibit lower responsiveness to the dexamethasone suppression test (DST), which tests GR mediated negative feedback onto HPA activity (Holsboer-Trachsler et al., 1991). Dexamethasone was also found to be less effective in suppressing immune activity in patients with depressive disorders (Maes et al., 1994). Taken together, GR function is modified in MDD, and patients develop what is termed GC resistance. Given that the actions of GR influence multiple systems in the body, GC resistance could serve as a link between MDD and immune dysregulation (Quan et al., 2003; Silverman and Sternberg, 2012).

HPA hyperactivity may be sufficient to cause depressive moods, since patients with Cushing's syndrome, characterized by hypersecretion of cortisol, often exhibit depressive symptoms (Starkman et al., 1981). In rodent models, administration of corticosterone can also elicit depressive-like behavior, and this effect is blocked by spironolactone, an MR antagonist (Wu et al., 2013). Since MR is associated with baseline GC actions, the increased secretion of cortisol in MDD patients may be in part mediating the disorder via MR activation. HPA dysregulation indeed presents itself to be integral to depressive symptoms. However, blocking GC signaling is not feasible due to its widespread effects on cardiovascular, reproductive, metabolic and immune function. Thus, recent attention has been turned toward specific upstream modulators of HPA activity in stress and MDD. Out of these investigations, the immune component appears to be the most likely target due to the overlaps between immune activation, the monoamine system, and the HPA axis.

### Acute TLR4 activation triggers the HPA axis

HPA activity is thought to mediate the immune involvement in MDD. Using LPS, previous research has been able to show that TLR4 signaling is able to stimulate the HPA axis (Mohn et al., 2011). Further studies showed that TLR4 activation is sufficient to cause GC release from adrenal cells (Vakharia and Hinson, 2004; Kanczkowski et al., 2013). TLR4 activation can also cause CRH gene upregulation in paraventricular neurons in the hypothalamus (Loum-Ribot et al., 2006), as well as increased CRH in serum (Goebel et al., 2011). Pituitary cells stimulated by LPS also produce ACTH, but there is contrasting data on whether this effect is CRH dependent (Elenkov et al., 1992; Mehet et al., 2012).

Despite clear evidence that innate immune activation via TLR4 can strongly stimulate the HPA axis, the exact mechanism is still unclear, as it is not known if TLR4 can directly modulate neuronal activity, or cause steroidogenesis via intracellular signaling. Part of the problem is, as illustrated earlier, where in the system LPS has the most effect, since it appears that TLR4 is present and can trigger increased secretion of CRH, ACTH, and GC. The other part of the problem lies in the many levels of immune activation that can also influence neuroendocrine activity. The following section reviews how TLR4 signaling can trigger the HPA axis and it's implications for MDD.

#### Cytokines and HPA activity

Cytokines are secreted by immune competent cells as a result of innate immune stimulation, including TLR4 activation by various ligands. As previously stated, cytokine administration is sufficient to cause sickness behavior. Cytokines can also upregulate HPA axis signaling on multiple levels through two main ways: (1) through reducing negative feedback on HPA signaling, and (2) by directly stimulating HPA activation. IL-1β, IL-6, and TNF-α are able to reduce the efficacy of GR, thus disinhibiting GR induced negative feedback on HPA activity (Pace et al., 2007; Bogaert et al., 2010). This effect could be due to protein-protein interactions with GR, which enables pro-inflammatory cytokines to influence GR translocation, ligand binding affinity and GR binding to GRE within the nucleus (for review, see Pace and Miller, 2009).

Secondly, cytokines can amplify the feed-forward signaling within the HPA axis. IL-6 has been shown to potentiate CRH activation of ACTH (Mehet et al., 2012), while IL-1β and stress have an additive effect on CRH secretion from the PVN (Chover-Gonzalez et al., 1993). Interestingly, peripheral and central IL-1β is upregulated in response to acute stress within 1 h, and 3 h after stress onset, respectively, and could therefore endogenously prime the HPA response. Moreover, this study also found that the administration of an IL-1β receptor antagonist diminished ACTH responses to restraint stress (Gądek-Michalska et al., 2011), showing that cytokines may partially mediate stress induced HPA activation. Given that an increase in peripheral IL-1β and circulating cortisol is observed in patients with MDD (Maes et al., 1991), this permissive effect on ACTH secretion could be in part driving the dysregulation between the immune and HPA secretion in MDD.

#### TLR4-related COX-2 production as part of steriodogenesis pathway

Besides cytokine interactions, COX-2 has also been shown to mediate TLR4 involvement in modulation of HPA activity. COX-2, an enzyme upregulated by TLR4 activation, catalyzes arachidonic acid into prostaglandin E2, which is part of the steroidogenesis pathway. COX-2 is therefore involved in the synthesis of GC at the level of the adrenal, and is shown to mediate LPS induced steroidogenesis within adrenal cells (Vakharia and Hinson, 2004). Furthermore, pharmacologically blocking COX-2 systemically is able to inhibit restraint stress induced GC both *in vivo* (Mouihate et al., 2010; Ma et al., 2013), and *in vitro* (Martinez Calejman et al., 2011). The effects of COX-2 extend beyond the adrenal gland, as expression of COX-2 in the PVN is important for sympathetic activation in response to restraint stress (Yamaguchi et al., 2010). In addition, COX-2 inhibition can influence higher order functions, buffering the effects of immobilization stress by reduced anxiety behavior and improve locomotor functioning and learning (Kumari et al., 2007).

#### Long-term effects of TLR4 activity on HPA axis function

Not only can TLR4 activation result in short-term stimulation of the HPA axis, but TLR4 can also influence HPA activity long after the stressor is resolved. For example, a single LPS challenge during early-life is sufficient to hyper-sensitize the CRH and ACTH response to both subsequent LPS and restraint stress when tested in adulthood, without baseline HPA differences when compared to vehicle controls in a rodent model (Mouihate et al., 2010). Early-life TLR4 activation also results in an increase in anxiety behavior during adulthood (Sominsky et al., 2013), thus fundamentally changing the stress response system. This suggests that TLR4 activity during developmentally sensitive periods may shape the HPA system, priming the system toward hyper-reactivity, and may even be changing individual predisposition toward stress-related disorders.

A good way of investigating the developmental consequence of TLR4 is through the use of genetic knockout models. TLR4 knockout mice are observed to have different HPA phenotypes when compared to match wild-type C57Bl/6 counterparts, showing increased adrenal gland volume and correspondingly higher baseline circulating glucocorticoid levels (Zacharowski et al., 2006). The direction of this change is not universally found however, as preliminary findings from our laboratory in Balb/c background TLR4 knockout mice have yielded opposite results. We have found that TLR4 genetic knockout mice have smaller adrenal glands as well as lower circulating GC (unpublished). Additionally, circulating ACTH was also elevated in mice lacking TLR4 when compared to matched controls.

Despite the differences in adrenal size and baseline ACTH levels, we found no difference between wild type and TLR4 knockout mice, in terms of adrenal responsiveness to ACTH administration in the same study (unpublished). Our findings support observations that systemic rather than adrenal MyD88 expression is important in regulating HPA activity (Kanczkowski et al., 2013). Taken together, these results suggest that although the TLR4 pathway can influence HPA activity, the innate immune system has little direct impact on adrenal function, and the observed HPA differences are likely to be driven by mechanisms within the CNS instead.

## Stress and systemic immunity

It is becoming clear that mood disorders such as depression have both immune and neuroendocrine components. Through interactions with the neuroendocrine system and central immune signaling, TLR4 is central to the physiological responses to immune stressors as well as baseline HPA activity. Yet at the same time, GCs classically suppress the immune system including the TLR4 pathway. When GCs bind to GR, immune suppression can occur in two main ways. Firstly, GR can directly interact with transcription factors NF-κB and AP-1 through protein-protein interactions, and in the process interfere with the pro-inflammatory transcription (Ratman et al., 2013). Following nuclear translocation, GR can also dimerise and bind to GRE on DNA, upregulating transcription of anti-inflammatory or repressing inflammatory genes such as IL-6 receptor gene (Muzikar et al., 2009). Both mechanisms appear important in driving anti-inflammatory actions of GCs as DNA binding appears important for the resolution of high-dose LPS induced inflammatory and behavioral response (Silverman et al., 2013), while GR can interfere with NF-κB induced transcription of proinflammatory genes (Novac et al., 2006). GR not only functions on the intracellular level, but can also downregulate macrophage expression of TLR4 mRNA in a dose and time dependent manner (Du et al., 2012). Given the varied roles of glucocorticoids in immune suppression, how does stress, which strongly triggers the HPA axis, cause increased immune signaling seen in MDD?

The answer likely lies in stress-induced adaptations, as recent evidence in rodent models has shown that stress itself can be both pro- and anti-inflammatory. Chronic footshock stress can induce bone-marrow derived monocytes infiltration into the hippocampus, thus increasing immune activity (Brevet et al., 2010). Acutely, footshock stress can also result in concurrent neuroendocrine and immune activation, characterized by increased hypothalamic IL-1β and TNF-α, adrenal IL-6, and COX-2, in addition to circulating ACTH and GC increase (Hueston and Deak, 2014). The authors also showed that injection of ACTH and CRH induced adrenal IL-6 and COX-2 mRNA expression, indicating that HPA activation can be pro-inflammatory. The increase in immune signaling seen in MDD may therefore be driven by stress itself.

The elevation of neurokine signaling appears stressor specific. Illustrating this, a meta-analysis showed that stress-induced IL-1β in the hypothalamus is most reproducible in footshock and immobilization stress models (Deak et al., 2005). On the other hand, social defeat stress increases prefrontal cortex IL-1β, IL-6, and TNF-α expression (Audet et al., 2011), in addition to increased monocyte infiltration (Wohleb et al., 2014). These variations in regional cytokine levels thus indicate a complex relationship between stressor type and the innate immune system within the CNS.

To reconcile the biphasic actions of HPA activation, in a recent review, Frank et al. (2013) argued that the timing of immune challenges and measurements is important in determining the direction of glucocorticoid actions. The authors proposed that glucocorticoids are anti- inflammatory during the stressor, but sensitizes the immune response after the stressor has ended, during what the “recovery phase” following the resolution of the stressor (Frank et al., 2013). Thus, timing of the “second-hit” as well as measurements of immune functioning following both stressors is therefore imperative to measuring GC actions on immune function. At this point, it is still unclear what mechanisms drive this biphasic effect, and how long this pro-inflammatory state persists following stress. In the following section, we review potential mechanisms, as well as present the case for TLR4 involvement in mediating the pro-inflammatory actions of the HPA system.

## Mechanisms of glucocorticoid induced pro-inflammatory responses

### Direct mechanisms through HPA activation

The HPA axis can directly influence immune signaling in two main ways, by reducing the inhibitory effects of glucocorticoid actions, or by directly stimulating the immune system. As mentioned in the previous section, there appears to be some form of GR adaptation, thus disrupting the actions of GR on the immune system and negative feedback onto the HPA system. This effect termed glucocorticoid resistance. Glucocorticoid resistance is predominantly thought to be due to either a reduced GR expression, or a selective reduction in GRα and a corresponding upregulation of GRβ, the inactive splice variant of the receptor that is unable to bind glucocorticoids (Silverman and Sternberg, 2012). Increased expression of pro-inflammatory cytokines correspond to elevated expression of GRβ, which could drive the disinhibition to immune response (Carvalho et al., 2014). Glucocorticoid resistance not only reduces glucocorticoid mediated immune suppression, but can in itself increase NF-κB responses in PBMC when exposed to glucocorticoids, thus reshaping the response to a previously anti-inflammatory stimulus (Dawson et al., 2012).

Contrary to classical actions, HPA activation can also directly trigger the immune response. CRH, which is secreted by PVN cells, may also directly stimulate the innate immune system. When exposed to CRH, mast cells have been shown to undergo degranulation, releasing cytokines into the extracellular space (Theoharides et al., 1995; Alysandratos et al., 2012). This directly implicates CRH in the increased central immune or neurokine signaling in stress-related disorders (Aguirre et al., 2013). On the other hand, CRH can also induce microglial apoptosis in the nanomolar range (Ock et al., 2006). It is therefore still unclear how the pro and anti-inflammatory effects are balanced in stress induced CRH release.

Pharmacological inhibition of GR reduces VCAM and ICAM expression in the microvasculature (Gregory et al., 2009), indicating the GR specific actions on immune cell migration. Indeed, low doses can be pro-inflammatory by stimulating phagocytosis and chemotaxis of macrophages via a GR mediated mechanism (Zhong et al., 2013). It has been previously established that GCs not only suppress the immune system, but at low doses directly induces production of macrophage migration inhibitory factor (MIF), a pro-inflammatory cytokine (Calandra et al., 1995). MIF can function as a chemokine, and stimulate CCL2 production when administered to the microvasculature, thus promoting migration of inflammatory cells to the site of damage (Gregory et al., 2009). Physiologically, MIF is involved in wound healing through promoting migration of endothelial progenitor cells to wounds (Grieb et al., 2012). Conversely, MIF is also implicated in the development of neuropathic pain (Alexander et al., 2012; Lerch et al., 2014). The exact reason for immune activation following the HPA response is still debated, but one of the more accepted reasons for this effect is that by increasing immune signaling, MIF constrains the HPA response in order to counteract glucocorticoid induced immune cell apoptosis. Through these mechanisms, it is thus possible for heightened HPA activity and immune activation to co-exist in patients with MDD.

### TLR4 mediated mechanisms of stress-induced pro-inflammatory response

#### TLR4 mediates immune priming effects of stress

Given the often-contradictory results on HPA function, the complexity of glucocorticoid signaling is becoming more appreciated. In order to explain the complex actions of glucocorticoids Sapolsky et al. (2000) proposed 4 categories of glucocorticoid action - permissive, inhibitory, excitatory and priming, encapsulating the different receptors activated, timing of response relative to stressors and type of tissue activated. Illustrating this, glucocorticoids are excitatory in terms of heart function but inhibit vascular function, cause the release of glucose aiding energy expenditure, yet also trigger stockpiling of fat in adipose tissue. At the same time, basal glucocorticoid expression can be permissive toward sympathetic activation of the adrenal medulla, thus influencing stress responses even before the HPA activation even occurs. Thus, glucocorticoids have different dose and time response relationships across different tissues, and their actions are dependent on basal or activated HPA states.

Recent studies are beginning to classify the priming or sensitizing effect of stress and HPA activity on immune function. Chronic variable and acute social disruptive stress can sensitize HPA and immune response to subsequent LPS challenge, differentially inducing larger neurokine and peripheral cytokine responses (Gibb et al., 2013). Repeated social defeat stress can also prime immune signaling in peripheral monocytes and dendritic cells in response to LPS, coupled with glucocorticoid resistance during the first 48 h after stressor, measured by immune cell expression following GC administration *in vitro* (Powell et al., 2009). Importantly, this immune priming is also seen in microglial populations 24 h after glucocorticoid treatment (Frank et al., 2011). Moreover, GC administration *in vivo* has been confirmed to emulate stress induced immune sensitization (Frank et al., 2010; Dey et al., 2014). This sensitizing effect was further blocked by a GR antagonist, indicating that GR signaling is essential (Frank et al., 2012). TLR2 and TLR4 activity could also be integral to glucocorticoid-induced immune priming in microglia, as administration of their respective antagonists prior to tailshock stress can prevent increased sensitivity in hippocampal tissues collected 24 h after the end of the stressor (Weber et al., 2013). Taken together, there appears to be crosstalk between the GR and TLR4 pathways, and both receptors appear to be important in driving immune cell sensitization and increased central immune signaling following stress.

The immune-priming effect of stress is proposed to mediate stress-induced side effects such as allodynia (Loram et al., 2011), and drug abuse (Frank et al., 2011), and could potentially be involved in MDD as well. PBMCs isolated from patients admitted for severe depressive episodes are more responsive to interferon-γ (IFN-γ) stimulation (Schlaak et al., 2012). Along with increased TLR4 mRNA and expression on PBMCs of patients with MDD (Kéri et al., 2014), the increased immune signaling in MDD patients could be indicative of a primed immune system, rather than chronic inflammation.

#### DAMPs released during stress cause TLR4 activation

DAMPs, or alarmins, are released endogenously from stressed, dead and dying cells as a signal for danger. They include HMGB1, various HSP, ATP, and Uric acid. During normal physiological activation, DAMPS have a non-inflammatory function within the cell. Conversely during situations of tissue damage, when released into the extracellular space, DAMPs alert the immune system to the damage in order to promote repair and direct traffic toward the damaged tissue, thus triggering the inflammatory response. This inflammatory response is in part driven by TLR4, since DAMPs including HMGB1 and various HSPs can activate the TLR4 pathway (Hutchinson et al., 2009; Laird et al., 2014). Furthermore, ATP can trigger innate immune signaling by activating a protein complex known as the inflammasome, which induces the maturation and release of cytokines IL-1β and IL-18 via a caspase-1 dependent mechanism (Chen et al., 2013). This effect is known to augment the inflammatory response to LPS, therefore amplifying TLR4 signaling (Ghonime et al., 2014).

DAMPS including HMGB1, uric acid and HSP72 are also released following tail-shock stress (Faraco et al., 2007; Maslanik et al., 2013), and thus are not limited to situations of tissue damage or cell death. DAMPs could therefore mediate the effect of stress on triggering or sensitizing the immune response, and this increased immune signaling may have wider implications for MDD. It is notable, however, that the mechanisms regulating the secretion of DAMPs in response to stress is not well characterized.

***High mobility group box 1***. HMGB1 functions as a chaperone protein within the cell by binding to proteins and transporting them between the cytoplasm and nucleus. During damage however, HMGB1 can be released into the extracellular space via an inflammasome mediated mechanism (Lu et al., 2012). Neural tissue is capable of releasing HMGB1 in response to glutamatergic excitotoxicity and glial activation as a result of LPS administration *in vitro* (Faraco et al., 2007). HMGB1 can also activate central immune signaling, as it can trigger TLR4 similar to LPS, via the MD2 and CD14 complex, and requires adaptor protein MyD88 to trigger downstream inflammatory actions (Kim et al., 2013). Moreover, psychological stress itself can induce an increase in HMGB1. For example, thymocytes are responsive to 15 min restraint stress, and release HMGB1 via GR signaling (Billing et al., 2012). There is further evidence showing that HMGB1 and GR can form complexes within the chromatin, increasing the residence time of GR when bound to DNA (Agresti et al., 2005). The functional consequence of this interaction, however, is still unclear. Given that HMGB1 is responsive to stress, interacts with GR, and is able to increase peripheral and central immune signaling, stress-induced immune sensitization through neurokine signaling could therefore be partially mediated by HMGB1.

HMGB1 can also cause an upregulation of Matrix metallopeptidase 9 (MMP9), an enzyme that results in the breakdown of the extracellular matrix (Qiu et al., 2010). Interestingly, increased MMP9 in circulation is associated with mood disorders such as depression and bipolar disorder (Domenici et al., 2010; Rybakowski et al., 2013). Together with evidence that MMP9 can also be upregulated as a consequence of microglial activity (Lively and Schlichter, 2013), MMP9 could be a possible result of stress induced HMGB1 upregulation and immune signaling within the CNS in MDD.

***Heat shock proteins***. HSPs were first discovered in the drosophila model to be produced in response to hyperthermia, and vary in protein weights ranging up to 110 kDA. HSP regulate the folding and unfolding of other proteins, and are released in response to cellular damage. Signaling danger, extracellular HSP can activate the innate immune system (Colaco et al., 2013). Within the HSP family, HSP70 and HSP90, the 70 and 90 kDa variants, are of most relevance to glucocorticoid and TLR4 signaling. HSP70 and HSP90 can bind TLR4, resulting in release of pro-inflammatory cytokines (Gong et al., 2009; Colaco et al., 2013). Furthermore, HSP90 also serves as a chaperone protein for TLR4, triggering endocytosis in response to ligand binding (Triantafilou and Triantafilou, 2004). Through these mechanisms, HSP90 plays an integral role in TLR4 signaling and in TLR4 related neuropathic pain (Hutchinson et al., 2009). Stress can also induce HSP expression, notably decreasing the ratio between GR and HSP70 and HSP90 expression in the hypothalamus (Simic et al., 2012).

Interestingly, HSP90 is a well-characterized chaperone protein for GR nuclear translocation and permits GC binding to GR (Ricketson et al., 2007). Although important for binding and translocation, Increased HSP90 expression can also impair GR function (Matysiak et al., 2008). This effect increases in chronic stress as compared to acute models of stress, and thus is proposed as one facet of glucocorticoid resistance. Stress induced changes in HSP can therefore either directly activate TLR4, change the trafficking TLR4 and GR, as well alter GR binding capacity. However, to what extent each of these mechanisms is involved, and the magnitude of the change has yet to be investigated in models of depressive-like behavior. Nevertheless, given the involvement in TLR4 and GR signaling, HSP could be an avenue for further research in linking stress and increased immune signaling in MDD.

#### TLR4 activation as a result of gut translocation of microbes

One way in which TLR4 ligands are upregulated by stress is through intestinal translocation. Recently, the role of gut microbiota in potentiating differences in mood and behavior is gaining traction within the literature (Hsiao et al., 2013). It has been hypothesized that stress can cause a disruption in intestinal tight junctions, which would increase translocation of microbiota into the system, thus inducing inflammatory responses. Indeed, GR appears to be involved in gut HSP70 upregulation and intestinal permeability in response to restraint stress (Ait-Belgnaoui et al., 2012, 2014).

There is some evidence showing that intestinal decontamination using orally administered antibiotics is able to block the tight junction disruption, as well as inflammatory and HPA responses to psychological stress (Gárate et al., 2011). Furthermore, antibiotic treatment is shown to mirror the ability of TLR4 antagonist in blocking stress induced depressive-like behavior (Gárate et al., 2011). This hypothesis is thus showing promise for developing new medication targeting the gut-brain axis in regulating behavior. On the other hand, the exact mechanisms of this gut-brain communication in respect to stress and MDD are unclear, since both ascending pathways and peripheral immune signaling could potentially be involved. In addition, the extent of intestinal translocation in the acute stress response requires more study, since it is not known if this effect is stressor specific.

## Conclusions

It is evident that the immune, neural and neuroendocrine systems are in constant multi-directional communication, and in the case of stress and MDD, patients exhibit a dysregulation of all three systems. Thus, the difficulty in finding treatment targets lies in untangling the multi-layered relationships. In the current review, we presented evidence centered on immune modulation of CNS and stress-induced adaptations observed in models of MDD. Stress is regarded as a necessary factor for the development of MDD through adaptations to the neuroendocrine and immune responses.

**Figure 1:**
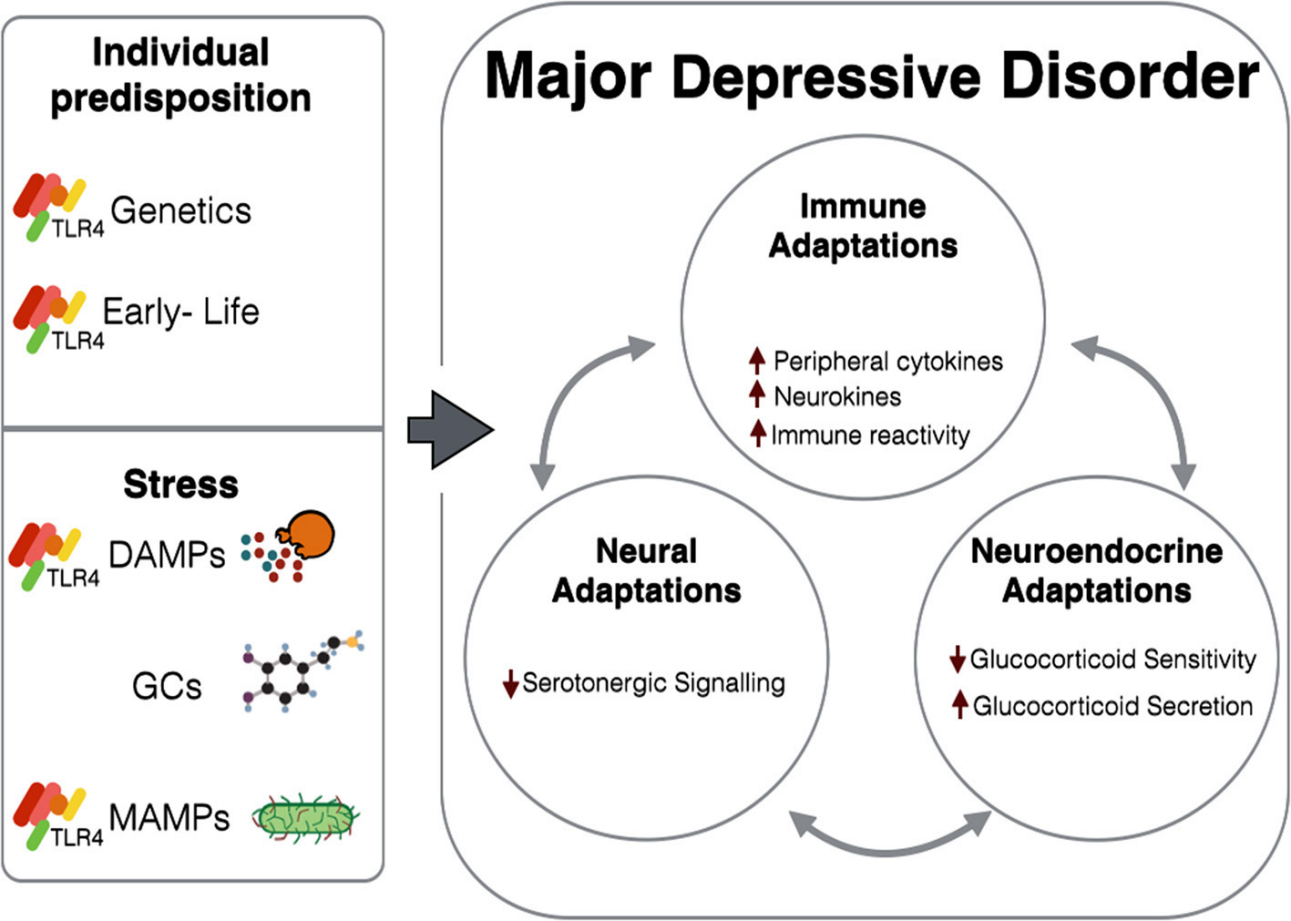
**Stress and individual predisposition combine to cause various immune, neuroendocrine, and neural adaptations observed in MDD**. Immune adaptations are at least in part attributed to TLR4-related mechanisms, either through direct activation by ligands (DAMPs and MAMPs) released as a result of stress, or by long-term impairment caused by genetics and early-life experiences. GCs can also interact with TLR4-dependent and GR-dependent mechanisms to induce systemic changes. In patients with MDD, these adaptations are not isolated to their respective systems, but instead further augment their deleterious effects through tri-directional communications.

Through use of knockout models as well as pharmacological agonists and antagonists, TLR4 activation has been shown to elicit depression-like symptoms in animal models both behaviorally and physiologically. Additionally, TLR4 could potentially mediate stress-induced immune signaling both in the periphery and within the CNS, as well as underlie stress-induced immune activity, through interactions with DAMPs, MAMPs, and GC signaling (Figure 1). TLR4, an innate immune receptor, could therefore be important in investigating the immune involvement in the pathophysiology of MDD.

On the other hand, this direct relationship between TLR4 and depression is still not fully understood, although timing and location of TLR4 activation appears to be important. The mode in which TLR4 influences MDD is not established, even though cytokines appear to be essential to the development of sickness behavior, other mechanisms such as direct interaction between TLR4 signaling pathway and other receptors (for example, GR) or intracellular signaling molecules could also play a role in the development of the disorder. Furthermore, due to the many compounds that can trigger TLR4 activation, an obvious question would be to identify which of those are most relevant to MDD. Critically, there is a need to move away from the use of LPS in the investigation of MDD, since it constrains the generalizability of conclusions to infectious factors. Although the immune system appears to be involved in the pathophysiology of MDD, it is unlikely that bacterial infections are the main factor, especially given that cytokines themselves can induce behavioral change in the absence of sickness. Instead, individual differences in immune activity could originate from alterations to immune signaling during critical periods during development, genetic disposition, or epigenetic changes that contribute to predispositions (Bilbo and Schwarz, 2009). Moreover, due to the sub-inflammatory and state driven nature of heightened immune signaling in MDD, endogenous mechanisms including DAMPs, neuroendocrine, neurogenic signals, or an increase in gut translocation of microbiomes are more likely involved. Thus, in the search for a more efficacious treatment of MDD, the impact on neural, neuroendocrine and immune systems must be considered within representative models of the disorder.

### Conflict of interest statement

The authors declare that the research was conducted in the absence of any commercial or financial relationships that could be construed as a potential conflict of interest.
